# Masticatory function in elderly compared to young adults

**DOI:** 10.1590/2317-1782/20212020364

**Published:** 2021-10-25

**Authors:** Verônica Fernandes Ramos, Anderson Francisco Silva, Melissa Picinato-Pirola

**Affiliations:** 1 Universidade de Brasília – UnB - Brasília (DF), Brasil.

**Keywords:** Aging, Aged, Mastication, Dental Prosthesis, Stomatognathic System, Envelhecimento, Idoso, Mastigação, Próteses Dentárias, Sistema Estomatognático

## Abstract

**Purpose:**

To characterize the masticatory function of the elderly and to compare total amount of time, masticatory strokes and total mastication score among the elderly and young adults.

**Methods:**

It is an observational, cross-sectional and analytical study. A total of 50 individuals participated, 25 elderly (mean age 66 years) and 25 young adults (mean age 22 years). The evaluation of mastication was performed by standardized filming of the usual mastication of a wheat flour biscuit. The masticatory type (alternated bilateral, simultaneous bilateral, preferential unilateral, chronic and anterior), masticatory score, total masticatory time and the total number of masticatory strokes were verified and compared between the elderly and young adults.

**Results:**

The predominant masticatory pattern in the young adults was the alternated bilateral mastication (52%), while, in the elderly, the simultaneous bilateral mastication predominated (48%). The use of dental prostheses had a significant influence on the total mastication score; elderly presented greater masticatory time and greater amount of masticatory strokes; however, the total masticatory score was lower for this group.

**Conclusion:**

The use of dental prosthesis has a significant influence on masticatory function. When compared to young adults, the elderly had a greater amount of time and masticatory strokes and a lower total mastication score.

## RESUMO

**Objetivo:**

Caracterizar a função mastigatória de idosos e comparar o total de tempo e golpes mastigatórios e o escore mastigatório total entre idosos e adultos jovens.

**Método:**

Trata-se de um estudo observacional, transversal e analítico. Um total de 50 indivíduos participaram do estudo, 25 idosos (idade média de 66 anos) e 25 adultos jovens (idade média de 22 anos). A avaliação da função mastigatória foi realizada por meio da filmagem padronizada da mastigação habitual de um biscoito maisena. O tipo mastigatório (bilateral alternado, bilateral simultâneo, unilateral preferencial, crônica e anterior), o escore mastigatório, o total do tempo mastigatório e o número total de golpes mastigatórios foram verificados e comparados entre os idosos e os adultos jovens.

**Resultados:**

O padrão mastigatório predominante nos adultos jovens foi a mastigação bilateral alternada (52%), enquanto que nos idosos predominou a mastigação bilateral simultânea (48%). O uso de prótese dentária apresentou influência significativa no escore total da mastigação; os idosos apresentaram maior tempo mastigatório e maior quantidade de golpes mastigatórios; contudo, o escore total da mastigação foi menor para este grupo.

**Conclusão:**

O uso de prótese dentária apresenta influência significativa na função mastigatória. Quando comparados aos jovens, idosos apresentam maior quantidade de tempo e golpes mastigatórios e menor escore total da mastigação.

## INTRODUCTION

The number of elderly people in the population increases over the years^(1)^ and aging causes several changes in the human body. Decreased strength and muscle mass occur due to changes in the intrinsic composition of the muscle fibers, as well as to the reduction of the size and quantity of them^(2)^. Elderly individuals have higher amounts of intramuscular fat when compared to young adults^(3)^ and increased fat infiltration also leads to the reduction of the muscle strength^(4)^.

Due to aging, morphophysiological changes occur in the orofacial structures, generating a decrease in the motor ability. They become insufficient and this leads to a reduction of the muscle tone of the phonoarticulatory organs^(5)^. In addition, factors such as reduction of the tension of the masticatory muscles, dental absences and bad adaptation of dental prostheses also contribute to alterations in these structures and in the stomatognathic functions, such as mastication^(6)^.

The systems of the human body undergo several changes due to normal aging, including the stomatognathic system^(7)^, and the orofacial structures undergo changes in mobility and muscle tone, thus, adaptations on masticatory function are necessary^(8)^.

The elderly perform a greater amount of masticatory cycles and take longer to masticate, leading to reduced masticatory performance^(9)^. These individuals perform adaptations of the masticatory function in order to reduce the damages resulting from aging^(8)^ such as the preference for soft foods^(7,8,10,11)^ and the installation of a simultaneous bilateral mastication pattern due to the reduction of tonus, incoordination of the masticatory muscles^(10)^ loss of natural teeth and bad adaptation of the dental prostheses^(8)^.

Elderly tend to have dental absences, because the older the age, the lower the number of natural teeth^(11)^. However, some elderly people with absent teeth make use of dental prostheses^(8,10,11)^, and it is important to consider that the health of the remaining teeth contribute positively to the masticatory function, since healthy natural teeth reduce the impact of aging in the mastication^(12)^. Considering that, currently, the objective is to prioritize the preservation of the natural teeth, it is important to develop programs with the aim of stimulating and guiding the preservation of the dental elements^(13)^.

After prosthetic rehabilitation, it is possible to observe improvement in masticatory performance, decreased time and masticatory cycles and less refusal of food^(14)^. Individuals who use removable partial prosthesis have satisfaction regarding the retention and adaptation of dental prosthesis, comfort and better performance of the masticatory function^(15)^. These advantages can also be observed in individuals who use dental implants, as well as satisfaction with factors associated with prosthesis aesthetics and oral hygiene^(16)^. However, regardless of the type of prosthesis, it is important its renewal and periodic maintenance, in order to improve the performance of stomatognathic functions, oral health and the comfort of the individual^(17)^.

With the accomplishment of the present study, it was possible to analyze the main characteristics of the masticatory function of the elderly and the factors that influence it, in order to know better the health of this population. It is understood that aging causes several changes and it is of the utmost importance to analyze this issue and reach real conclusions, so that prevention and speech therapist interventions measures can be taken to adapt the appropriated masticatory type and the strength of orofacial muscles, with the objective to offering a better quality of life during aging.

Thus, the objective of the present study was to characterize the masticatory function of the elderly and to compare the masticatory time, the number of masticatory strokes and the masticatory function among the elderly and young adults.

## METHODS

### Study sample and inclusion and exclusion criteria

It is an observational, cross-sectional, analytical study. All procedures carried out in studies involving human subjects were in accordance with the ethical standards of the Ethics in Research Committee of Faculdade de Ceilândia - Universidade de Brasília, according to legal advice number 2.380.411. All participants signed the Informed Consent Form. For the definition of the sample, it was performed the sample calculation and 25 participants in the control group (CG) and 25 in the experimental group (GI) were estimated, considering the sample error of 5% and the confidence interval of 95%, what resulted in a sample of 24 individuals in each group.

The power of the test was estimated in the GPower software using the multiple linear regression method from a R^2^ measure obtained from a sample. From the parameters described in Table 1, the power of the test for the study was estimated at 98%.

**Table 1:** Power of the test

**Parameter**	**Value**
R^2^	0.572
Effect size f^2^	1.336
Significance level (α)	0.05
Sample size	25
Number of predictors	5

A total of 90 individuals were recruited to participate in the study, but only 50 were enrolled in the inclusion criteria and accepted to participate in the study, 25 of them were of GI, 13 women and 12 men, with a mean age of 66.64 years and 25 of them of CG, 13 women and 12 men, with a mean age of 22.1 years.

For the GI, the adopted inclusion criteria were individuals aged 60 years and over, regardless of dental absences and/or independent of using partial or total dental prostheses, as well as dental implants. For the CG composition, the inclusion criteria were individuals aged between 18 and 36 years; without dental absences (except for third molars); Class I according to Angle's classification, evaluated through the analysis of the relationship between the first mandibular molar and the first maxillary molar, considering the sagittal axis^(18)^; no changes in facial structures or dental occlusion, independent to had used an orthodontic appliance, however, do not could using it at the moment of the evaluation; and without complaints of signs or symptoms of temporomandibular joint dysfunction.

For both groups, subjects who had motor disorders, intellectual difficulties, neurological alterations, who had toothache or orofacial pain and who had undergone some surgical procedure or had suffered some trauma in the head and neck region were excluded from the study. These data were evaluated by the investigators, that were speech therapist, by means of observational evaluation and during the anamnesis, through reports of the participants.

### Evaluation procedures

Data collection was performed at the Laboratory of Human Communication and Orofacial Functions of the Speech-Language Pathology course at Faculdade de Ceilândia – Universidade de Brasília.

At first, an anamnesis was carried out by means of a semistructured questionnaire applied by the researchers in order to verify if the individual met the inclusion or exclusion criteria and for the characterization of the sample.

To verify the nutritional status of the participants, the Body Mass Index (BMI) was calculated (BMI = weight(kg)/ height(m)^2^). For the calculation of BMI, data regarding weight and height were collected as reported by the participants themselves. It was considered eutrophic, the BMI value from 18.7 to < 25 kg/m^2^ ^(19)^.

The number of absent teeth in the GI was verified by counting the dental elements. The number of absent teeth without prosthetic rehabilitation was considered as dental absence. The type of dental prosthesis, when present, was also observed. In order to verify if the prosthesis was badly adapted, the participants were asked if the prosthesis hurt the area and/or if there was displacement of the prosthesis, according to the perception of the elderly. During the evaluation of the masticatory function, it was also observed if there was displacement of the prosthesis through the posture and contraction of the facial muscles and perception of difficulties to masticate.

During the evaluation, the participants sat in a reclined chair with their feet flat on the floor. They were filmed using a smartphone iPhone 7 model (Apple, California, USA), 4.7” screen and a 12MP camera. The device was positioned in front of the individual on a tripod, being adopted as standardization the distance of 1 meter between the lens of the device and the participant. The height of the tripod was adjusted so as to focus the entire face, neck and shoulders of the subject.

For the assessment of the masticatory function, the Orofacial Myofunctional Evaluation with Scores for Elders Protocol (OMES-ELDERS)^(20)^ was used for GI. For the CG, the Protocol of Orofacial Myofunctional Evaluation with Expanded Scores (OMES-E) was used^(21)^. Each participant was instructed to perform the usual free mastication of a Maizena® (wheat flour) biscuit (Marilan, Marília, São Paulo, Brazil). Through OMES-E, applied in the CG, it was observed the type of incision of the food (bites with incisors, canines, premolars or molars); the masticatory type, determined by counting the masticatory strokes that occurred on each side (left, right, simultaneous or anterior); and other behaviors and signs of change, considering movement of head or other parts of the body, altered posture and food leakage. The maximum score of the protocol in the mastication evaluation presented the total of 20 points. In the GI, evaluated using the OMES-ELDERS, the same criteria was considered, but the masticatory type was classified according to the presence or absence of prosthesis. The maximum protocol score in the mastication evaluation presented a total of 18 points. Because the GI maximum score consists of 18 points and the CG of 20 points, in order to compare the mean score obtained, the maximum score was considered as 100% and the mean score percentage reached by the CG was compared to the one reached by the GI.

Both GI and CG were evaluated for time during video analysis, with the aid of the same device used for the video recording. The chronometer was started when the food was placed in the mouth and finished after the last swallowing. To determine the total time, it was added the amount of seconds obtained in each portion of the food, being excluded the intervals between the last swallowing and the next bite of the biscuit. The amount of masticatory strokes was verified by analyzing the video, *i.e.*, all the times that occurred opening and closing movements of the mandible to grind the food were observed. At the end, it was added the amount of strokes performed in each portion of the food, so that it was possible to obtain the total number of the masticatory strokes.

To verify de concordance index was used the Kappa Coefficiente and the analysis of the filming was carried out by 2 trained and calibrated researchers. The concordance index was between 0.81-1.00 (almost perfect).

### Statistical analysis

The descriptive statistical analyzes were calculated for all variables and were expressed in frequency and percentage for the categorical variables and in mean, median and standard deviation for the numerical variables.

To verify the influence of gender, age, prosthesis use, time and number of masticatory strokes in the total mastication score of the GI, the multiple linear regression model was used. To compare total time, masticatory strokes and total masticatory score between GI and CG, the non-parametric Mann-Whitney test was used.

For the statistical analysis, the SAS 9.2 program was used. For the present study, it was considered the significance level of 5%.

## RESULTS

The description of the variables regarding age, total mastication score, amount of portions that the food was divided, total masticatory time, total amount of masticatory strokes and achieved percentage of the total masticatory score is showed in Table 2. The comparison of the total mastication score was also performed among elderly participants who used dental prosthesis and those who did not use it. As shown in Table 2, it was observed that the GI divided the food in greater quantity of portions. It was also found that elderly people who used dental prosthesis had a higher masticatory score when compared to those who did not use it. It is important to consider that in the present study all individuals who did not use dental prosthesis presented dental losses. According to the semistructured questionnaire, was observed that 24% of the GI has preference for soft foods and this wasn’t present on CG.

**Table 2:** Comparison between GI and CG regarding age, total mastication score, number of portions, total time and masticatory strokes and reached percentage of possible total masticatory score. Comparison of the mean of the total mastication score among elderly who used dental prosthesis and those who did not use it

**Variables**	**Groups**	**Mean**	**Median**	**Standard Deviation**
Age (years)	GI	66.64	66	5.25
	CG	22.1	22	3.26
BMI	GI	27.1	26.22	4.57
	CG	23	22.49	4.16
Total mastication score	GI (max.: 18)	14.76	17	3.06
	CG (max.: 20)	18.4	19	1.89
Number of portions	GI	3.04	3	0.84
	CG	2.9	3	0.73
Masticatory time	GI	52.68	56	14.12
	CG	36.6	35	9.11
Masticatory strokes	GI	61.8	64	18.41
	CG	42.6	42	12.1
Total mastication score (%)	GI	82	94.44	16.99
	CG	92.2	95	9.47
**Masticatory score in the use of dental prosthesis**				
Use of dental prosthesis	GI	15.5	17	2.5
Did not use dental prosthesis (presented dental losses)	GI	9.7	10	1.53

**Caption:** Max.: possibility of maximum score; GI: experimental group; CG: control group.

In relation to the amount of dental elements, a total of 64% of the GI had dental absences, with a mean of 2.8 absent teeth. No elderly had total dental absence without some type of prosthetic rehabilitation and 36% of the GI did not have any absent dental elements, because they used dental prosthesis to supply all absent teeth.

The use of dental prosthesis was present in 88% of the GI, and the type of most used prosthetic rehabilitation by GI was the dental implant (40%) and 8% had badly adapted prostheses. Even with the use of prosthesis, 52% of the GI still had absent teeth. However, 12% did not use dental prostheses and all of them presented dental absences.

The predominant masticatory pattern in the CG was the alternated bilateral mastication (52%), while in the GI, the simultaneous bilateral mastication was predominant (48%). Elderly with prosthesis displacement had simultaneous bilateral mastication as a masticatory type and all the GI participants (100%) presented tension of the facial musculature during mastication.

Table 3 shows the influence of gender, age, use of dental prostheses, total time and masticatory strokes in the total mastication score achieved by GI. It was verified that the use of dental prosthesis influenced the total mastication score. In other words, the elderly who use prosthetics had higher mean values of mastication scores, as shown in Table 2.

**Table 3:** Influence of gender, age, use of dental prostheses, total masticatory time and total masticatory strokes in the total mastication score

Parameters	Estimation	Standard Error	CI 95%		p Value
Gender (f - m)	-2.0197	6.5351	-15.6978	11.6584	0.76
Age	-0.4173	0.5748	-1.6204	0.7858	0.48
Use or not of dental prothesis	31.6382	9.4220	11.9177	51.3586	<0.01 *
Total masticatory time	-0.1706	0.2996	-0.7976	0.4565	0.58
Total masticatory strokes	-0.2228	0.2157	-0.6742	0.2286	0.31

R-square = 0.572

Test used for statistical analysis: multiple linear regression model.

* p≤0.05 statistically significant

**Caption:** f: female; m: male; CI: confidence interval.

**Explanatory note for CI:** With 95% confidence, the interval 11.9177 to 51.3586 contains the true estimation for the influence of the use of dental prosthesis on masticatory function.

In the comparison of total masticatory time, masticatory strokes and of the percentage of total possible masticatory score, a significant difference was found, with GI showing a greater amount of masticatory strokes and longer mastication time in relation to CG. However, the percentage of the total mastication score for this group was lower (Table 4).

**Table 4:** Comparison between the GI and CG groups of the total amount of masticatory strokes, total masticatory time and total mastication score

	**Groups**	**Mean**	**Median**	**Standard Deviation**	**p Value**
Total of masticatory strokes	**GI CG**	61.8	64	18.41	<0.01 *
		42.64	42	12.1	
Total masticatory time	**GI CG**	52.68	56	14.12	<0.01 *
		36.6	35	9.11	
Total masticatory score	**GI CG**	82	94.44	16.99	0.01 *
(% of the total possible)		92.2	95	9.47	

Test used for statistical analysis: Mann-Whitney test

* p≤0.05 statistically significant

## DISCUSSION

The present study favored the understanding of physical and anatomophysiological aspects of the elderly. The simultaneous bilateral mastication pattern was present in most of the GI, whereas in CG there was a predominance of alternated bilateral mastication. This finding demonstrates that elderly perform adaptations of the masticatory function by means of vertical movements of the mandible, being in accordance with other studies^(8,10)^. These adaptations may be associated with the reduction of masticatory muscles tonicity, dental absences, bad adaptation of dental prostheses and preference for soft consistencies^(6)^. In this study, it was observed that many of the elderly participants evaluated had dental prostheses as well as a preference for soft consistencies.

More than half (64%) of the population studied had dental absences. This datum corroborates the findings of other studies, demonstrating that the amount of natural teeth decreases with age^(6,11)^. A study carried out in Japan^(22)^ showed that elderly with higher numbers of natural teeth have a better quality of life, with lower mortality rates and longer life expectancy. The study by Ikebe et al.^(23)^ demonstrated that the elderly participants in the study had a greater number of natural teeth and a lower prevalence of dental prostheses use, but this assumption was not observed in this study, being relevant for the Brazilian population to increase oral health care through development of oral promotion and rehabilitation programs^(24)^.

With the objective of filling dental absences, many elderly undergo prosthetic rehabilitation. In the present study, 88% of the individuals use dental prosthesis, being in agreement with the findings of the literature^(6,8,10,11,16,23)^. Dental implants are the most used form of prosthetic rehabilitation by GI. Different data were found in the literature, being the total removable prosthesis the most used by the populations studied^(8)^. However, dental implants have aesthetic and functional benefits^(16)^ and provide better performance of the masticatory function, due to the stability during mastication, when compared to total removable prosthesis^(25)^.

In the present study it was possible to observe that only the use of dental prostheses showed a positive influence on mastication. In other words, the mastication score was higher in individuals who used dental prosthesis. This datum corroborates the literature findings, confirming that after prosthetic rehabilitation there is greater masticatory performance^(14-16,25)^. However, the study by Ayres et al.^(26)^ found that prosthetic rehabilitation causes changes in the stomatognathic system, with an impact on mastication, because the individuals who undergo this process have reduced facial muscle tonus, when compared to individuals who do not use dental prosthesis. Only 12% of the GI did not use any type of dental prosthesis. However, all of them had absent dental elements. It is important to consider that, in individuals with large number of absent teeth, the prosthetic rehabilitation will improve the masticatory function, because a greater number of posterior occlusal contacts will favor the grinding of food^(23)^.

It was found that gender and age did not significantly influence the overall mastication score. This datum corroborates the findings of the study by Ikebe et al.^(23)^, justifying that age alone does not lead to masticatory changes, but the adjacent impacts to aging do, such as tooth losses, for example. Thus, with a good conservation of the dental health one can maintain a better masticatory performance.

When comparing with CG, it was found that GI presented a greater total amount of time and masticatory strokes, but a lower mastication score. Is known that the elderly have a reduce of the number and the size of the motor units of the orofacial muscles^(2)^, besides the presence of intramuscular fat^(3)^. Thus, will occur a reduction of tonus and strength of the masticatory muscles^(9)^ and a consequent increase of time and masticatory strokes. Besides that, the dental absences too has an influence, because when the number of occlusal contacts are reduced, a greater amount of masticatory strokes are necessary to grind the food and the masticatory time is higher^(27)^. Considering that 64% of the GI present dental absences, it can be justified the increase of time and masticatory strokes in the elderly when compared to the young ones. Thus, the reduction of masticatory function occured due to these alterations, and the total amount of time and masticatory strokes did not have a significant influence on the total mastication score, according to data found in the present study. Other factor that has influence on mastication is the salivary flow, considering that an adequate quantity of saliva is essential to a better masticatory performance^(23)^.

A randomized study found that despite the increase of the masticatory strokes, the amount of food to be ingested by the elderly will not be altered and this is an important factor for maintaining the nutritional status of the elderly^(28)^. This datum corroborates the findings of the present study, because, even with a greater amount of time and masticatory strokes, the BMI mean of the GI is within the normality pattern for the elderly.

The literature shows that after prosthetic rehabilitation there is reduction of masticatory time and strokes and better masticatory performance^(14)^. Besides, the study by van Kampen et al.^(29)^ found that after rehabilitation with dental implants there is better performance of masticatory function and reduction of masticatory time and masticatory strokes. These findings confirm data found in the present study, because the use of prosthesis had a positive influence on masticatory function and the dental implant was used by 40% of the GI. This may justify a better masticatory performance in elderly who had undergone prosthetic rehabilitation.

According to Lepley et al.^(30)^, the shorter the masticatory time and the fewer the masticatory strokes performed, the better the mastication performance. This finding is in accordance with the data of the present study, justifying the fact that GI presented a lower mastication score and consequent greater time and masticatory strokes when compared to the CG.

The present study presented as obstacles the difficulty to find individuals who were willing to participate in the research and who fit the inclusion criteria, mainly to the CG, being necessary recruit a large quantity of individuals to find the participants of the study. Despite this difficulty, the number of participants in the study was sufficient to prove the findings, as demonstrated by the sample calculation. It is a study that contributed to a greater clarification about the influences of variables on the masticatory function and changes due to aging.

## CONCLUSION

The masticatory function is influenced by the use of dental prostheses in elderly individuals. Greater mastication time, greater amount of masticatory strokes and lower masticatory score were observed in the elderly when compared to young adults.
